# INSTAPALS: FACILITATING INTERGENERATIONAL CONTACT THROUGH TECHNOLOGY

**DOI:** 10.1093/geroni/igz038.2978

**Published:** 2019-11-08

**Authors:** Ashley Lytle, Nancy Nowacek

**Affiliations:** Stevens Institute of Technology, Hoboken, New Jersey, United States

## Abstract

Using the traditional framework of Pen Pals, Instapals was a project that facilitated 1-to-1 intergenerational relationships through daily exchanges on Instagram for 30 days. Although communication channels have exploded in the past 10 years in large part to social platforms and digital technologies, the diversity of daily social interactions has decreased. More and more, society has become siloed by age, interest, and belief. Building off intergroup contact theory, Instapals was designed to encourage positive intergenerational contact between younger (undergraduate students) and older adults (individuals 65+) and challenge ageist beliefs. Intergenerational social exchanges occurred both on Instagram and during three in-person meetings. Among undergraduate students, attitudes and stereotypes toward older adults and aging were assessed at three timepoints (before meeting their older adult partner, during a mid-point evaluation, and at the end of the project). Quantitative analyses demonstrate a decrease in aging anxiety, a decrease in psychological concerns about the aging process, and a greater endorsement of positive perceptions toward one’s older adult partner. Qualitative analyses of written responses from students (collected before meeting their older adult partner and at the end of the project) revealed that the Instapals project helped students challenge ageist attitudes and stereotypes and was perceived to be a positive experience overall. Implications suggest that positive intergenerational contact can be facilitated and enhanced through the use of technology. Future research should explore whether attitudes, stereotypes, and self-perceptions of aging changed among older adults as well as other technological mechanisms for facilitating intergenerational contact.

